# Assessing citation networks for dissemination and implementation research frameworks

**DOI:** 10.1186/s13012-017-0628-2

**Published:** 2017-07-28

**Authors:** Ted A. Skolarus, Todd Lehmann, Rachel G. Tabak, Jenine Harris, Jesse Lecy, Anne E. Sales

**Affiliations:** 1 Center for Clinical Management Research, VA Ann Arbor Healthcare System, Ann Arbor, MI 48105 USA; 2 Dow Division of Health Services Research, Department of Urology, University of Michigan, Ann Arbor, MI 48109 USA; 3 Urology Section, VA Ann Arbor Healthcare System, Department of Urology, University of Michigan, Ann Arbor, MI 48109 USA; 4 Department of Political Science, College of Literature, Science and the Arts, University of Michigan, Ann Arbor, MI 48109 USA; 5 Prevention Research Center in St. Louis/George Warren Brown School of Social Work at Washington University in St. Louis, St. Louis, MO 63130 USA; 6 Maxwell School of Citizenship and Public Affairs, Syracuse University, Syracuse, NY 13244 USA; 7 Department of Learning Health Sciences, University of Michigan Medical School, University of Michigan, Ann Arbor, MI 48109 USA

**Keywords:** Network analysis, Knowledge translation, Management science, Model, Implementation science, Bibliometrics, Quality improvement, Behavioral theory

## Abstract

**Background:**

A recent review of frameworks used in dissemination and implementation (D&I) science described 61 judged to be related either to dissemination, implementation, or both. The current use of these frameworks and their contributions to D&I science more broadly has yet to be reviewed. For these reasons, our objective was to determine the role of these frameworks in the development of D&I science.

**Methods:**

We used the Web of Science™ Core Collection and Google Scholar™ to conduct a citation network analysis for the key frameworks described in a recent systematic review of D&I frameworks (Am J Prev Med 43(3):337–350, 2012). From January to August 2016, we collected framework data including title, reference, publication year, and citations per year and conducted descriptive and main path network analyses to identify those most important in holding the current citation network for D&I frameworks together.

**Results:**

The source article contained 119 cited references, with 50 published articles and 11 documents identified as a primary framework reference. The average citations per year for the 61 frameworks reviewed ranged from 0.7 to 103.3 among articles published from 1985 to 2012. Citation rates from all frameworks are reported with citation network analyses for the framework review article and ten highly cited framework seed articles. The main path for the D&I framework citation network is presented.

**Conclusions:**

We examined citation rates and the main paths through the citation network to delineate the current landscape of D&I framework research, and opportunities for advancing framework development and use. Dissemination and implementation researchers and practitioners may consider frequency of framework citation and our network findings when planning implementation efforts to build upon this foundation and promote systematic advances in D&I science.

**Electronic supplementary material:**

The online version of this article (doi:10.1186/s13012-017-0628-2) contains supplementary material, which is available to authorized users.

## Background

The field of dissemination and implementation (D&I) science continues to evolve with contributions from a variety of disciplines, researchers, and institutions across the globe [1]. Significant advances in our understanding of how to conceptualize D&I research and practice were facilitated by a recent comprehensive review of relevant models, theories, and frameworks [2]. The review identified 61 frameworks to guide D&I researchers and practitioners in their research-to-practice activities at different socio-ecologic levels within the health care system (individual, organization, community, healthcare system, policy). The goal was to develop a D&I framework inventory to inform selection efforts for researchers and practitioners based on a given framework’s construct flexibility, its predilection for dissemination and/or implementation activities, as well as its socio-ecologic level targeting.

However, better understanding the most frequently cited D&I frameworks and the citation networks surrounding these frameworks can also provide useful information for selection, conceptualization, and resources for operationalization. For example, in cases where several different frameworks might be applicable to a given implementation intervention, identifying the most prominent and commonly applied frameworks in the field could have several advantages. First, it could provide researchers and practitioners with the most supporting literature to inform their effort. Second, accessing this information may increase the chances of intervention success and therefore help the best frameworks emerge. Third, as the framework literature evolves, there will be increasing opportunities to advance D&I science with respect to fidelity of framework use, core framework components, standardized measurement, advantages and disadvantages of a given framework, and ultimately implementation outcomes [3]. More broadly, mapping D&I framework networks can build upon this foundation to promote systematic advances in D&I science through identifying the common set of assumptions and knowledge that constitutes consensus in the field.

Bibliometric (or citation) analysis is one method to investigate the scholarly landscape surrounding D&I frameworks from the review. This quantitative technique is increasingly applied to measure the impact of academic research and examine relationships using tools such as citation network analysis [4–6]. In general, citation network analysis provides a map of the most highly cited publications within a given research domain, much like the way Google™ uses page rank to identify the most relevant websites [7]. This approach to understanding the state of scientific advancement has been used across a range of fields, including public administration, public health service systems, physical activity environments, and analytic method development, to discern the degree to which information flows through a scholarly network and identify opportunities for transdisciplinary collaboration and crosstalk [8–14]. Using citation analysis to examine the rapidly evolving D&I field could not only indicate the most frequently cited D&I frameworks but also determine their relationships across time and discipline, and map the emerging knowledge network constituting the D&I framework field.

For these reasons, we conducted a citation network analysis of D&I research frameworks. We created a snapshot of the scientific development of D&I framework research based on carefully selected framework articles followed forward in time as they integrated into the growing body of D&I knowledge. We examined citation rates and the main paths through the citation network to delineate the current landscape of D&I framework research, and opportunities for advancing framework development and use.

## Methods

### Citation network analysis

We used a citation data network collection tool, the Citation Network Analyzer (CNA), to generate the data and conduct our study [15, 16]. This tool uses a constrained snowball sampling approach to identify a network of documents (i.e., journal and conference papers, theses and dissertations, academic books, pre-prints, abstracts, technical reports) in Google Scholar™ that can be used for descriptive, main path, and other network analyses via an R software package. In general, a constrained snowball sample of academic publications is created by identifying seed articles, determining the levels of data (articles that cite the seeds, articles that cite those, and so on), and selecting the sampling rate at each level. This vetted, efficient and inclusive networking approach to following citations forward in time is uniquely suited to advance our current understanding of the literature surrounding D&I framework development and use. In addition, the output from the CNA tool can be used to graphically represent the citation network and assign weights to the articles based on their importance in maintaining the network architecture as described below.

Our approach of using citation network analysis to conduct structured literature reviews was based on prior work using the CNA tool [9, 10, 13, 15, 17]. This approach can lead to a less biased assessment of the academic literature than traditional narrative reviews for at least two reasons. First, a citation analysis approach can avoid the cognitive bias associated with traditional literature searches using keyword searches which may be limited by the researcher expertise, training, and preferences. Second, the use of Google Scholar™ and a snowball sampling technique based on selected seed articles, rather than Web of Science™ citation tools based on keywords for instance, is able to survey a broader scope of publications that may be relevant to D&I frameworks especially given their expansive roots in fields ranging from agriculture, business, and political science to public health and medicine [18, 19]. In addition, the CNA tool allows for a constrained approach to snowball sampling, rather than traditional snowball sampling where the sample grows exponentially, in order to limit the articles at each level from the seed article to arrive at empirical findings using a fraction of the data [15].

As detailed in Additional file 1 *,* we conducted two analyses using this novel approach. First, we synthesized the literature covered in the framework review article by Tabak et al. [2] with respect to recent citations and performed a structured literature review of the article itself. Next, we applied a structured literature review to a snowball sample of ten framework articles identified as the most important by the study team, largely based on the Tabak review. Overall, this work allowed us to understand the relevance of the framework review article as a D&I resource and to identify those frameworks forming the current backbone of the D&I framework field (i.e., framework articles in the network’s main path).

#### Characterizing the Tabak et al. framework review article and its citation network

The Tabak systematic review contained 119 references, with 50 published articles and 11 documents (reports/chapters/books) identified as a primary D&I framework reference (*n* = 61) [2]. These D&I frameworks were identified first through selecting commonly cited frameworks, then through snowball sampling and expert consultation including with U.S. National Institute of Health offıcials who process and review D&I grants. Frameworks were excluded from the review according to the following criteria: (1) focused on practitioner rather than D&I researcher; (2) applied to individual behavior change only (i.e., without ties to local, organizational or community dissemination); (3) intended only for national level use versus local, community, or organizational level; (4) frameworks focused only on dissemination after research study completion; and (5) articles not written in the English language. The frameworks were then judged by the authors to be related either more to dissemination, implementation, or both equally. Each framework’s construct flexibility was rated as broad and flexible versus operational and defined for a given context and activity. Last, the socio-ecologic level (individual, organization, community, healthcare system, policy) targeted by the framework was categorized, with most operating at more than one level.

We extracted the primary citation for each framework. In cases where more than one primary reference was used (*n* = 21), we selected the most relevant reference, usually the oldest, as the primary reference. The primary references for 11 frameworks were reports, chapters, or books. Because peer-reviewed articles were the most common documents cited in this study, we use the term *article* to denote all documents throughout the remainder of the manuscript.

To better understand the framework articles discussed in the Tabak review, we conducted descriptive analyses to identify the most common journals, authors, and countries of origin for the 61 models. We also examined the citation rates for each framework. We defined a citation rate as the number of citations/year(s) since publication. We used the Web of Science™ Core Collection in January 2016 to conduct these descriptive citation analyses and inform our subsequent network analysis described in the Additional file 1 *.*

#### Citation network analysis of selected D&I frameworks

Next, we conducted a citation network analysis of ten carefully selected D&I framework articles we felt reflected the current state of the field. Eight of these were based on citation rates and the Tabak review. However, we also included two additional frameworks given their relevance to implementation science and relatively high citation rates: (1) Theoretical Domains Framework (TDF) [20, 21] and the (2) Knowledge to Action Framework (KTA) [22], for a total of ten seed articles for our next citation network analysis. Both of these models were developed by researchers outside the USA and were not included in the Tabak review. The details of the D&I framework citation analysis are included in the Additional file 1 *.*

Last, we performed a main path analysis to identify the connectedness and links among the articles considered to be the backbone of the D&I framework citation network. This approach identifies the key articles influencing D&I models based on the selected seed articles. We determined the traversal weights indicating the proportion of network paths that included a given article node in the network [23]. For instance, a traversal weight of 0.25 for framework X indicates that its article exists in 25% of the citation paths in the network. This traversal weight indicates the importance of any particular node (i.e., article) in the network. We constructed the main path by removing all ties in the network scoring below the 95% percentile for traversal weight value. We normalized the traversal weights according to flow using the Search Path Count method [24]. All computations were accomplished with Pajek [23].

All analyses were conducted between January 2016 and August 2016. This study was deemed not regulated by the Institutional Review Board at the University of Michigan.

## Results

### Tabak framework review article and its citation network

**Fig. 1:**
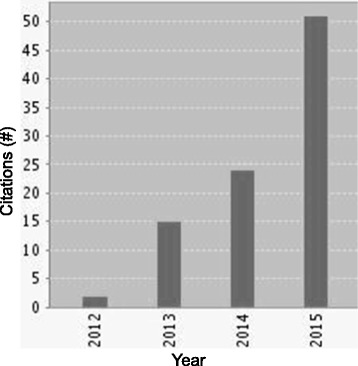
Citation report through 2015 for ‘Bridging Research and Practice Models for Dissemination and Implementation Research’ by Tabak et al. [2]

As illustrated in Fig. 1, the Tabak framework review article is an increasingly cited resource. As of January 2016, it had been cumulatively cited 456 times across 388 articles and other source items indexed within Web of Science™ Core Collection. As shown in Table 1, there was a broad distribution of citation numbers and annual citation rates across the 61 framework articles within the Tabak review and our two selected framework articles (KTA and TDF). The average number of citations per year ranged from 0 to 1949 among articles published from 1962 to 2012. The outlier with the highest citation rate was a book reference for Rogers’ Diffusion of Innovations.

**Table 1:** Citations for D&I frameworks in published articles as of January 2016

No.	Framework—manuscripts	Web of Science™ Core Collection Citations	Citations/year	Year	Country	Most relevant framework reference, usually oldest
-	^a^ *Knowledge to Action Framework* ^**b**^	1445	160.6	2006	Canada	Graham ID, Logan J, Harrison MB, et al. Lost in knowledge translation: time for a map? J Contin Educ Health Prof. 2006 Winter;26(1):13–24 [22].
1	^a^A Conceptual Model for the Diffusion of Innovations in Service Organizations	1136	103.3	2004	UK	Greenhalgh T, Robert G, Macfarlane F, et al. Diffusion of innovations in service organizations: systematic review and recommendations. Milbank Q 2004;82(4):581–629 [27].
2	^a^Sticky Knowledge	1949	102.6	1996	USA	Szulanski G. Exploring internal stickiness: impediments to the transfer of best practice within the fırm. Strat Manag J 1996;17:27–43 [37].
-	^a^ *Theoretical Domains Framework* ^**b**^	613	61.3	2005	UK	Michie S, Johnston M, Abraham C, et al. “Psychological Theory” Group. Making psychological theory useful for implementing evidence based practice: a consensus approach. Qual Saf Health Care. 2005 Feb;14(1):26–33 [38].
3	^a^The RE-AIM Framework	731	45.7	1999	USA	Glasgow RE, Vogt TM, Boles SM. Evaluating the public health impact of health promotion interventions: the RE-AIM framework. Am J Public Health 1999;89(9):1322–7 [30].
4	^a^Consolidated Framework for Implementation Research	257	42.8	2009	USA	Damschroder LJ, Aron DC, Keith RE, et al. Fostering implementation of health services research fındings into practice: a consolidated framework for advancing implementation science. Implement Sci 2009;4:50 [28].
5	^a^Conceptual Model of Evidence-Based Practice Implementation in Public Service Sectors	118	29.5	2011	USA	Aarons GA, Hurlburt M, Horwitz SM. Advancing a conceptual model of evidence-based practice implementation in public service sectors. Adm Policy Ment Health 2011;38(1):4–23 [31].
6	^a^Conceptual Model of Implementation Research	147	24.5	2009	USA	Proctor EK, Landsverk J, Aarons G, et al. Implementation research in mental health services: an emerging science with conceptual, methodological, and training challenges. Adm Policy Ment Health 2009;36(1):24–34 [29].
7	^a^Implementation Effectiveness Model	426	22.4	1996	USA	Klein KJ, Sorra JS. The challenge of innovation implementation. Acad Manag Rev. 1996:1055–80 [26]
8	^a^Promoting Action on Research Implementation in Health Services	379	22.3	1998	UK	Kitson A, Harvey G, McCormack B. Enabling the implementation of evidence based practice: a conceptual framework. Qual Health Care 1998;7(3):149 [25].
9	Research Knowledge Infrastructure	263	21.9	2003	Canada	Lavis JN, Robertson D, Woodside JM, et al. How can research organizations more effectively transfer research knowledge to decision makers? Milbank Q 2003;81(2):221–48 [39].
10	Interactive Systems Framework	143	20.4	2008	USA	Wandersman A, Duffy J, Flaspohler P, et al. Bridging the gap between prevention research and practice: the interactive systems framework for dissemination and implementation. Am J Comm Psych 2008;41(3–4):171–81 [40].
11	Utilization-Focused Surveillance Framework	110	18.3	2009	USA	Green LW, Ottoson JM, Garcia C, et al. Diffusion theory and knowledge dissemination, utilization, and integration in public health. Annu Rev. Public Health 2009;30:151–74 [41].
12	Normalization Process Theory	98	16.3	2009	UK	May C, Finch T. Implementing, embedding, and integrating practices: an outline of normalization process theory. Soc J Br Soc Assoc 2009;43(3):535–54 [42].
13	Multi-level Conceptual Framework of Organizational Innovation Adoption	177	13.6	2002	Netherlands	Frambach RT, Schillewaert N. Organizational innovation adoption: a multi-level framework of determinants and opportunities for future research. J Business Res 2002;55(2):163–76 [43].
14	Davis’ Pathman-PRECEED Model	211	11.1	1996	USA	Pathman DE, Konrad TR, Freed GL, et al. The awareness-to-adherence model of the steps to clinical guideline compliance: the case of pediatric vaccine recommendations. Med Care 1996;34(9):873 [44].
15	Pronovost’s 4E’s Process Theory	76	10.9	2008	USA	Pronovost PJ, Berenholtz SM, Needham DM. Translating evidence into practice: a model for large scale knowledge translation. BMJ 2008;337:a1714 [45].
16	Knowledge Exchange Framework	64	10.7	2009	UK	Ward V, House A, Hamer S. Developing a framework for transferring knowledge into action: a thematic analysis of the literature. J Health Serv Res Policy 2009;14(3):156–64 [46].
17	Framework of Dissemination in Health Services Intervention Research	73	10.4	2008	USA	Mendel P, Meredith LS, Schoenbaum M, et al. Interventions in organizational and community context: a framework for building evidence on dissemination and implementation in hlth srvcs rsrch. Adm Pol Ment Hlth 2008;35(1–2):21–37 [47].
18	A Framework for Analyzing Adoption of Complex Health Innovations	52	10.4	2010	UK	Atun, Rifat, de Jongh, Thyra, Secci, Federica, et al. Integration of targeted health interventions into health systems: a conceptual framework for analysis. HEALTH POLICY AND PLANNING Vol: 25, Iss: 2 Pgs: 104–111, MAR 2010 [48]
19	Pathways to Evidence Informed Policy	102	10.2	2005	Australia	Bowen S, Zwi AB. Pathways to “evidence-informed” policy and practice: a framework for action. PLoS Med 2005;2(7):e166 [49].
20	Availability, Responsiveness & Continuity (ARC): An Organizational & Community	92	9.2	2005	USA	Glisson C, Schoenwald SK. The ARC organizational and community intervention strategy for implementing evidence-based children’s mental health treatments. Ment Health Serv Res 2005;7(4):243–59 [50].
21	Practical, Robust Implementation and Sustainability Model (PRISM)	54	7.7	2008	USA	Feldstein AC, Glasgow RE. A practical, robust implementation and sustainability model (PRISM) for integrating research fındings into practice. Jt Comm J Qual Patient Saf 2008;34(4):228–43 [51].
22	An Organizational Theory of Innovation Implementation	43	7.2	2009	USA	Weiner BJ, Lewis MA, Linnan LA. Using organization theory to understand the determinants of effective implementation of worksite health promotion programs. Health Educ Res 2009;24(2):292–305 [52].
23	Ottawa Model of Research Use	103	6.1	1998	Canada	Logan J, Graham ID. Toward a comprehensive interdisciplinary model of health care research use. Sci Commun 1998;20(2):227 [53].
24	Policy Framework for Increasing Diffusion of Evidence-Based Physical Activity Interventions	54	6.0	2006	Australia	Owen N, Glanz K, Sallis JF, Kelder SH. Evidence-based approaches to dissemination and diffusion of physical activity interventions. Am J Prev Med 2006;31(4S):S35–S44 [54].
25	Replicating Effective Programs Plus Framework	43	5.4	2007	USA	Kilbourne AM, Neumann MS, Pincus HA, et al. Implementing evidence-based interventions in health care: application of the replicating effective programs framework. Implement Sci 2007;2:42 [55].
26	Framework for Knowledge Translation	62	5.2	2003	Canada	Jacobson N, Butterill D, Goering P. Development of a framework for knowledge translation: understanding user context. J Health Serv Res Policy 2003;8(2):94–9 [56].
27	Collaborative Model for Knowledge Translation Between Research and Practice Settings	33	4.7	2008	Canada	Baumbusch JL, Kirkham SR, Khan KB, et al. Pursuing common agendas: a collaborative model for knowledge translation between research and practice in clinical settings. Res Nurs Health 2008; 31(2):130–40 [57].
28	A Convergent Diffusion and Social Marketing Approach for Dissemination	34	3.8	2006	USA	Dearing JW, Maibach EW, Buller DB. A convergent diffusion and social marketing approach for disseminating proven approaches to physical activity promotion. Am J Prev Med 2006;31(4S):S11–S23 [58].
29	Framework for the Dissemination & Utilization of Research for Health-Care Policy & Practice	47	3.6	2002	Canada	Dobbins M, Ciliska D, Cockerill R, et al. A framework for the dissemination and utilization of research for health-care policy and practice. Online J Knowl Synth Nurs 2002;9:7 [59].
30	Push–Pull Capacity Model	32	3.6	2006	USA	Green LW, Orleans CT, Ottoson JM, et al. Inferring strategies for disseminating physical activity policies, programs, and practices from the successes of tobacco control. Am J Prev Med 2006;31(4S):S66 –S81 [60].
31	Critical Realism & the Arts Research Utilization Model (CRARUM)	17	2.8	2009	Canada	Kontos PC, Poland BD. Mapping new theoretical and methodological terrain for knowledge translation: contributions from critical realism and the arts. Implement Sci 2009;4:1 [61].
32	Coordinated Implementation Model	62	2.8	1993	Canada	Lomas J. Retailing research: increasing the role of evidence in clinical services for childbirth. Milbank Q 1993;71(3):439–75 [62].
33	Knowledge Translation Model of Tehran University of Medical Sciences	17	2.4	2008	Iran	Majdzadeh R, Sadighi J, Nejat S, Mahani AS, Gholami J. Knowledge translation for research utilization: design of a knowledge translation model at Tehran University of Medical Sciences. J Cont Educ Health Prof 2008;28(4):270–7 [63].
34	Dissemination of Evidence-based Interventions to Prevent Obesity	6	2.0	2012	USA	Dreisinger ML, Boland EM, Filler CD, Baker EA, Hessel AS, Brownson RC. Contextual factors influencing readiness for dissemination of obesity prevention programs and policies. Health Educ Res 2012;27(2):292–306 [64].
35	OPTIONS Model	33	1.9	1998	Canada	Martin GW, Herie MA, Turner BJ, Cunningham JA. A social marketing model for disseminating research-based treatments to addictions treatment providers. Addiction 1998;93(11):1703–15 [65].
36	Conceptualizing Dissemination Research and Activity: Canadian Heart Health Initiative	23	1.9	2003	Canada	Elliott SJ, O’Loughlin J, Robinson K, et al. Conceptualizing dissemination research and activity: the case of the Canadian Heart Health Initiative. Health Educ Behav 2003;30(3):267–82; discussion 283–6 [66].
38	Conceptual Framework for Research Knowledge Transfer and Utilization	22	1.8	2003	Canada	Kramer DM, Cole DC. Sustained, intensive engagement to promote health and safety knowledge transfer to and utilization by workplaces. Sci Commun 2003;25(1):56 [67].
37	“4E” Framework for Knowledge Dissemination and Utilization	22	1.8	2003	USA	Farkas M, Jette AM, Tennstedt S, Haley SM, Quinn V. Knowledge dissemination and utilization in gerontology: an organizing framework. Gerontologist 2003;43(S1):47 [68].
39	Linking Systems Framework	18	1.8	2005	Canada	Robinson K, Elliott SJ, Driedger SM, et al. Using linking systems to build capacity and enhance dissemination in heart health promotion: a Canadian multiple-case study. Health Educ Res 2005;20(5): 499–513 [69].
40	Blueprint for Dissemination	9	1.8	2010	USA	Yuan CT, Nembhard IM, Stern AF, Brush JE Jr., Krumholz HM, Bradley EH. Blueprint for the dissemination of evidence-based practices in health care. Issue Brief (Commonw Fund) 2010;86:1–16 [70].
41	Health Promotion Research Center Framework	5	1.7	2012	USA	Harris JR, Cheadle A, Hannon PA, et al. A framework for disseminating evidence-based health promotion practices. Prev Chronic Dis 2012;9:E22 [71].
42	A Framework for Spread	16	1.6	2005	USA	Nolan K, Schall MW, Erb F, et al. Using a framework for spread: The case of patient access in the Veterans Health Administration. Jt Comm J Qual Patient Saf 2005;31(6):339–47 [72].
43	Model for Locally Based Research Transfer Development	25	1.6	1999	Canada	Anderson M, Cosby J, Swan B, et al. The use of research in local health service agencies. Soc Sci Med 1999; 49(8):1007–19 [73].
44	A Six-Step Framework For International Physical Activity Dissemination	14	1.6	2006	Australia	Bauman AE, Nelson DE, Pratt M, et al. Dissemination of physical activity evidence, programs, policies, and surveillance in the international public health arena. Am J Prev Med 2006;31(4S):S57–S65 [74].
45	CDC DHAP’s Research-to-Practice Framework	23	1.5	2000	USA	Sogolow ED, Kay LS, Doll LS, et al. Strengthening HIV prevention: application of a research-to-practice framework. AIDS Educ Prev 2000;12(5S):21–32 [75].
46	Health Promotion Technology Transfer Process	28	1.5	1996	USA	Orlandi MA. Health promotion technology transfer: organizational perspectives. Can J Public Health 1996;87(S2):S28 –S33 [76].
47	RAND Model of Persuasive Communication and Diffusion of Communication and Medical Innovation	31	1.0	1985	USA	Winkler JD, Lohr KN, Brook RH. Persuasive communication and medical technology assessment. Arch Intern Med 1985;145(2):314–7 [77].
48	A Conceptual Model of Knowledge Utilization	21	1.0	1993	USA	Lester JP. The utilization of policy analysis by state agency offıcials. Sci Commun 1993;14(3):267 [78].
49	Model for Improving the Dissemination of Nursing Research	19	0.7	1989	USA	Funk SG, Tornquist EM, Champagne MT. A model for improving the dissemination of nursing research. West J Nurs Res 1989;11(3): 361–72 [79].
50	Effective Dissemination Strategies	9	0.7	2002	UK	Scullion PA. Effective dissemination strategies. Nurs Res 2002; 10(1):65–77 [80].
	Frameworks – Other documents					
51	Diffusion of Innovation		1144.2	1962	USA	Rogers, Everett M. (1962). Diffusion of Innovations. Glencoe: Free Press. ISBN 0–612–62,843-4 [81]
52	Streams of Policy Process		395.8	1984	USA	Kingdon JW. Agendas, alternatives, and public policies. Boston: Little, Brown, 1984 [82]
53	Active Implementation Framework		213.9	2005	USA	Fixsen DL, Naoom SF, Blasé KA, et al. Implementation research: a synthesis of the literature. Tampa FL: University of South Florida, Louis de la Parte Florida Mental Health Institute, The National Implementation Research Network, 2005 [83].
54	The Precede–Proceed Model		73.8	2005	USA	Green LW, Kreuter MW. Health program planning: an educational and ecological approach. 4th ed. New York: McGraw-Hill, 2005 [84].
55	Research Development Dissemination and Utilization Framework		22.7	1969	USA	Havelock RG. Planning for innovation through dissemination and utilization of knowledge. Centre for Research on Utilization of Scientifıc Knowledge, Institute for Social Research, University of Michigan, 1969 [85].
56	Real-World Dissemination		14.7	1992	UK	Pettigrew AM, Ferlie E, McKee L. Shaping strategic change: making change in large organizations: the case of the National Health Service. Thousand Oaks CA: Sage Publications, 1992 [86].
57	A Framework for the Transfer of Patient Safety Research into Practice		3.5	2005	USA	Nieva VF, Murphy R, Ridley N, et al. From science to service: a framework for the transfer of patient safety. 2005. In: Henriksen K, Battles JB, Marks ES, Lewin DI, eds. Advances in patient safety: from research to implementation (Vol. 2: Concepts and methodology). Rockville MD: Agency for Healthcare Research and Quality, 2005 [87].
58	Framework for Dissemination of Evidence-Based Policy		1.0	2012	USA	Dodson EA, Brownson RC, Weiss SW. Policy dissemination research. In: Brownson R, Colditz G, Proctor EK, eds. Dissemination and implementation research in health: translating science to practice. Oxford: Oxford University Press, 2012 [88].
59	Marketing and Distribution System for Public Health		1.0	2012	USA	Kreuter MW, Casey CM, Bernhardt JM. Enhancing dissemination though marketing and distribution systems: a vision for public health. In: Brownson RC, Colditz G, Proctor EK, eds. Dissemination and implementation research in health: translating science to practice. New York: Oxford University Press, 2012 [89].
60	Facilitating Adoption of Best Practices (FAB) Model		0.1	2008	USA	Damush T, Bravata DM, Plue L, et al. Facilitation of Best Practices (FAB) Framework. Stroke QUERI Center annual report. 2008 [90].
61	Interacting Elements of Integrating Science, Policy, and Practice		0.0	2011	USA	TIDIRH Working Group. Interacting elements of integrating science, policy, and practice. In: Training institute for dissemination and implementation research in health. Conference proceedings. Chapel Hill NC, 2011 [91].

^a^Included as one of ten seed articles for citation network analysis

^**b**^Two additional frameworks were included along with the Tabak framework review articles given their relevance to implementation science - Theoretical Domains Framework (TDF) [20] and the Knowledge to Action Framework (KTA) [22]

**Fig. 2:**
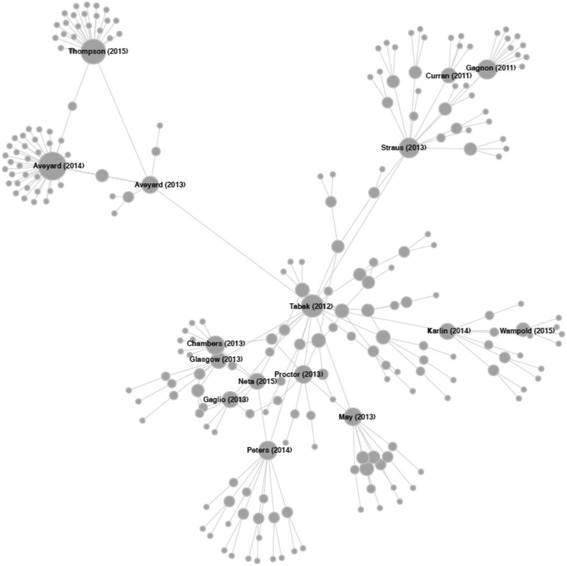
Citation network for ‘Bridging Research and Practice Models for Dissemination and Implementation Research’ by Tabak et al. [2]. Most first authors contributed only one article (one = 177). Those authors with two articles—Aarons, G; Archambault, P; Bjurlin, M; Blease, CR; Brownson, R; Chambers, D; Chor, K; Davidoff, F; Edwards, N; Gagliardi, A; Kozica, S; May, C; Naci, H; Neta, G; Page, A; Partridge, SR; Rhoades, E; Trevithick, P; Trockel, M; three articles—Aveyard, H; O’Brien, J; Proctor, E; four articles—Glasgow, R and Powell, B; and six articles—Thompson, N

Based on the structured literature review of the Tabak article using the CNA tool, we identified 239 articles across the network and its three levels of ‘distance.’ This included 17 level-one articles directly referencing the Tabak article, with the remainder of articles residing two and three levels from the Tabak source article. The majority of the documents were journal articles (84%), followed by books (16%). The articles in the Tabak network were published between 2002 and 2016, with 51 articles published prior to the source article year of 2012. The majority (86%) of these were three levels from the Tabak seed article and (35%) were book references. We identified 202 unique first authors contributing to this network. Each author contributed 1.18 articles (standard deviation (SD) = 0.58), on average. Most first authors contributed only one article to the network (one = 177; two = 19, three = 3, four = 2, six = 1). We identified 123 unique journals (books excluded) contributing to the Tabak network, each providing an average of 1.62 articles (SD = 2.63). Most journals contributed one article (*n* = 95). The top three journals producing the most articles were: *Implementation Science* (*n* = 29), *Annual Review of Public Health* (*n* = 6), and *BMC Public Health* (*n* = 5). All other journals had four or fewer articles each. The articles in the Tabak network were cited between 0 and 4410 times. The top ten cited articles in the Tabak network are shown in Table 2, and none of which served as a primary framework reference. As illustrated in Fig. 2, there were prominent ties in the Tabak network to social care and the law by Aveyard; normalization process and general implementation theory by May; implementation work by Glasgow, Proctor, Neta, and Chambers; a gateway to broader literature via a movement science article by Peters; a Karlin article which ties in psychotherapy; and a 2013 contribution by Straus that was an introduction to knowledge translation in healthcare.

**Table 2:** Ten most cited articles within the Tabak framework review citation network

Title	First author	Year	Journal	Google Scholar Citations	Network Level
Doing your research project: a guide for first-time researchers [92]	Bell	2014	Book	4410	3
Research methods for sports studies [93]	Gratton	2010	Book	734	3
The utilization of health research in policy-making: concepts, examples and methods of assessment [94]	Hanney	2003	*Health Research Policy and Systems*	558	3
Information retrieval: a health and biomedical perspective [95]	Hersh	2008	Book	446	3
Anti-oppressive practice: social care and the law [96]	Dalrymple	2006	Book	377	3
Doing a literature review in health and social care: A practical guide [97]	Aveyard	2014	Book	341	2
Social work skills: a practice handbook [98]	Trevithick	2005	Book	318	3
Understanding social work: preparing for practice [99]	Thompson	2015	Book	236	2
Reflexivity, its meanings and relevance for social work: a critical review of the literature [100]	D’Cruz	2007	*British Journal of Social Work*	220	3
Synthesis of recommendations for the assessment and management of low back pain from recent clinical practice guidelines [101]	Dagenais	2010	*The Spine Journal*	213	3

### Citation network analysis of selected D&I frameworks

The citation network for our seed articles highlighted in Table 1 included 355 unique documents published between 1996 and 2014. There were 302,472 citation links connecting the articles in this network. The majority of citations was from 323 journal articles (91%), followed by 29 books (8%), and 3 in-proceedings (1%). We identified 274 unique first authors, each contributing 1.30 articles (SD = 0.84), on average. The majority of first authors provided one article to the network with only six authors contributing greater than three. We also identified 128 unique journals contributing to this network, each providing an average of 2.52 articles (SD = 4.04). While many journals contributed one article (*n* = 29), the top five journals producing the most articles were: *Strategic Management Journal* (*n* = 29), *Academy of Management Journal* (*n* = 25), *Implementation Science* (*n* = 20), *Organization Science* (*n* = 15), and *Management Science* (*n* = 10). All other journals contributed less than ten articles each. The top ten cited articles are shown in Table 3, with Szulanski’s Sticky Knowledge as the only primary framework reference from the Tabak review. The remainder of articles tended to focus on business practices and knowledge sharing, collaboration networks, and social and/or intellectual capital. The articles for the D&I framework network contributed between 64 and 12,680 citations, with a median of 489.

**Table 3:** Ten most cited articles within the D&I framework citation network

Title	First author	Journal	Year	Google Scholar Citations
Social capital, intellectual capital, and the organizational advantage [102]	Nahapiet	*Academy of Management Review*	1998	12,680
Dynamic capabilities: what are they? [103]	Eisenhardt	*Strategic Management Journal*	2000	10,085
The relational view: Cooperative strategy and sources of interorganizational competitive advantage [104]	Dyer	*Academy of Management Review*	1998	9681
Cultivating communities of practice: A guide to managing knowledge [105]	Wenger	Book	2002	8548
Review: Knowledge management and knowledge management systems: Conceptual foundations and research issues [106]	Alavi	*MIS quarterly*	2001	8166
Exploring internal stickiness: Impediments to the transfer of best practice within the firm [37]	Szulanski	*Strategic Management Journal*	1996	7694
Absorptive capacity: A review, reconceptualization, and extension [107]	Zahra	*Academy of Management Review*	2002	6194
The search-transfer problem: The role of weak ties in sharing knowledge across organization subunits [108]	Hansen	*Administrative Science Quarterly*	1999	5528
Collaboration networks, structural holes, and innovation: A longitudinal study [109]	Ahuja	*Administrative Science Quarterly*	2000	4140
Creating and managing a high performance knowledge-sharing network: the Toyota case [110]	Dyer	*Strategic Management Journal*	2000	3509

**Fig. 3:**
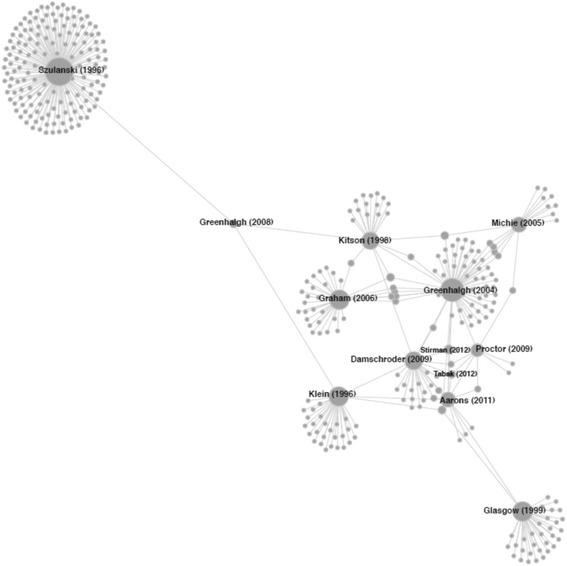
D&I framework citation network. The majority of first authors provided only one article to the network with only six authors contributing greater than three including Hansen, M and Pronovost, P—four articles; Michie, S and Rycroft-Malone, J—five articles; Greenhalgh, T—seven articles; and Glasgow, R—eight articles

**Fig. 4:**
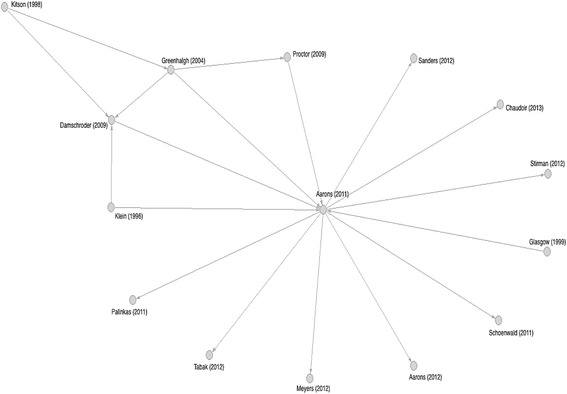
The main path for a D&I framework citation network. A simple interpretation of the citation network main path is that these articles are the most important in holding the entire D&I framework citation network together. In this case, seven of the ten D&I framework seed articles were part of the main path, along with eight non-seed articles

As illustrated in Fig. 3, the D&I framework citation network appears centered around the 2004 Greenhalgh et al. article with prominent ties to the Theoretical Domains Framework, the Knowledge to Action Framework, the Promoting Action on Research Implementation in Health Services Framework (PARiHS), the Consolidated Framework for Implementation Research (CFIR), and an article conceptualizing implementation outcomes, among others. A more complete picture of the network’s primary core is offered with the main path analysis, which consists of those ties above the 95% percentile score for traversal weight (0.0106). The main path, illustrated in Fig. 4, is comprised of the 15 articles listed in Table 4. A simple interpretation of the main path is that these articles are most important in holding the entire D&I framework citation network together. In this case, seven of the ten D&I framework seed articles are part of the main path, along with eight non-seed articles. Visually, one can inspect the main path and observe the chronological flow of influence from earlier to more recent publications. Kitson [25] and Klein [26] act as the primary originating sources of influence in the main path, which serve to influence Greenhalgh [27], Damschroder [28], and Proctor [29]. These five articles, along with Glasgow [30], all converge in Aarons [31], which acts as a major hub for the remainder of the more recent works on the periphery of the main path.

**Table 4:** Main path articles for leading D&I research frameworks

Network vertex^a^	Seed article	Traversal weight	Author/year/article
10	Yes	0.34	Kitson et al. 1998. “Enabling the implementation of evidence based practice: a conceptual framework” [25]
1	Yes	0.24	Greenhalgh et al. 2004. “Diffusion of innovations in service organizations: systematic review and recommendations” [27]
6	Yes	0.18	Damschroder et al. 2009. “Fostering implementation of health services research findings into practice: a consolidated framework for advancing implementation science” [28]
9	Yes	0.14	Klein and Sorra. 1996. “The challenge of innovation implementation.” [26]
7	Yes	0.12	Aarons et al. 2011. “Advancing a conceptual model of evidence based practice implementation in public service sectors” [31]
3	Yes	0.09	Glasgow et al. 1998. “Evaluating the public health impact of health promotion interventions: the RE-AIM framework.” [30]
8	Yes	0.03	Proctor et al. 2009. “Implementation research in mental health services: an emerging science with conceptual, methodological, and training challenges.” [29]
54	No	0.03	Stirman et al. 2012. “The sustainability of new programs and innovations: a review of the empirical literature and recommendations for future research” [111]
293	No	0.02	Tabak et al. 2012. “Bridging research and practice: models for dissemination and implementation research” [2]
297	No	0.02	Meyers et al. 2012. “The quality implementation framework: A synthesis of critical steps in the implementation process” [112]
300	No	0.02	Chaudoir et al. 2013. “Measuring factors affecting implementation of health innovations: a systematic review of structural, organizational, provider, patient, and innovation level measures” [3]
309	No	0.02	Schoenwald et al. 2011. “Toward the effective and efficient measurement of implementation fidelity” [113]
310	No	0.02	Palinkas et al. 2011. “Mixed method designs in implementation research” [114]
311	No	0.02	Sanders. 2012. “Development, evaluation, and multinational dissemination of the Triple P Positive Parenting Program” [115]
312	No	0.02	Aarons et al. 2012. “The organizational social context of mental health services and clinician attitudes toward evidence based practice: a United States national study” [116]

^a^Network vertex is a designated point in the network where 1 through 10 indicates a seed article

## Discussion

Using citation analysis, we identified the most frequently cited D&I frameworks and their relationships across time and discipline and mapped the knowledge network constituting the D&I framework field. We discovered that the Tabak framework review has been increasingly cited and that it was included in the periphery of the main D&I framework network path indicating its value as a recognized resource for D&I researchers and practitioners. We identified the leading journals and authors contributing to the D&I framework literature using methods that limit cognitive biases associated with traditional literature searches using keywords. Using the CNA tool to conduct our structured literature review, we were able to identify the main path articles that signify those most important in holding the entire D&I framework citation network together. Overall, D&I researchers and practitioners may consider frequency of citation and this network structure when planning implementation efforts to build upon this foundation and promote systematic advances in D&I science. Further work is necessary to delineate how these frameworks are being used in the literature, framework selection criteria for planning D&I research efforts, the core components of these frameworks, and how framework use relates to improved implementation outcomes [3].

This study provides insight into at least two aspects of the evolving D&I scientific field. First, it confirms that D&I research has witnessed a surge of frameworks with most developed in the last two decades [2]. However, we found that the majority of articles were rarely cited, leaving only a few highly cited frameworks. It is difficult to know whether more recent frameworks will be used or not based on this analysis though several recent articles, including the Tabak review, were highly cited. Nonetheless, there does appear to be framework saturation creating an increasing need to delve further into better understanding the current cadre rather than creating new D&I frameworks. Second, taking into consideration citation rates and this network structure may be a key factor to consider when choosing a framework, in addition to the socioecological level, construct flexibility, and location on the D&I spectrum. For example, increasing citations and centrality in the network indicates more literature is available to highlight the advantages and disadvantages of using a given framework. In addition, there may be more operational and measurement resources with increasing centrality. Taking these additional aspects into consideration creates opportunities to scrutinize frameworks, starting with those in the main path, and advance D&I science by examining issues of fidelity, core and adaptive components, measurement, and relationships to implementation outcomes [1].

We found a broad range of scientific fields contributing to the D&I citation network given our use of Google Scholar™ and its extensive search capabilities [7, 19]. This reinforces the need to scan literature outside of health-related fields to discover new guidance for D&I sciences. For example, other than the specialized journal *Implementation Science*, which focuses specifically on the field, most citations of the Tabak framework review article were from public health journals due in part to it being a narrative review that used snowball sampling methods and focused on health. In addition, the journals other than *Implementation Science,* which published the highest number of citations in the broader D&I framework network, were all in the management and business fields. This is consistent with a prior review of leading management journals that found a significant degree of knowledge translation and organizational change literature relevant to D&I in healthcare [32]. While there is some current cross-over among these fields, they are often quite distinct and separate from each other when it comes to research and practice. Taken together, our findings suggest that greater efforts to scan across these journals and fields could provide unique transdisciplinary collaborations and innovation opportunities to hasten D&I research and practice. For that matter, D&I advances could also serve to improve management and business practices.

However, citing a framework does not imply use or specify what its application entails. How to operationalize determinants of practice across frameworks also needs to be better understood to advance D&I science. A recent study examined use of the KTA framework using citation analysis and systematic review to see if the framework was used in practice and how [33]. The authors found that it was used with varying degrees of completeness from a simple reference to integration into the design, delivery, and evaluation of the implementation activities. The latter contributing most to advancing D&I science and generalizability of outcomes. Similarly, another recent systematic review examined use of the CFIR among empirical studies in the peer-reviewed literature [34]. Twenty-six articles met inclusion criteria across a breadth of settings and units of analysis. Justification for which CFIR constructs were selected, integration throughout the research study, and relation to outcomes remained poorly articulated, again limiting contributions to D&I research more broadly. Furthermore, systematic efforts to reconcile determinants of healthcare professional practice across 12 different frameworks have generated practical checklists and implementation strategy recommendations to support implementation and quality improvement efforts [35]. Better understanding framework use, consolidation and operationalization of framework determinants, not just citations, could yield more to consider when selecting and using D&I frameworks for research and practice.

There are several limitations to our study approach. First, framework citation rates are influenced by a multitude of factors including journal impact factor, the authors’ fame and publication rate, the degree of research in a given field, whether citation is perceived as positive or negative, and do not necessarily indicate the quality of a given publication or framework [5–7, 19]. Nonetheless, citation rates do serve as an approximation of the impact of a scholarly work. We also used an expert-led review article for seed article identification and a robust network analysis tool, coupled with citation rate data, to provide our snapshot of the scientific development of the D&I framework field with substantial face validity. Second, there could be issues with respect to language and the definition of D&I research leading to ascertainment bias. Using our comprehensive CNA approach in Google Scholar™, rather than keyword searches for example, actually created a broader scope for our study. Last, whether the use of highly cited documents (e.g., textbooks) as seed articles, rather than the journal articles selected as seeds in our study, would dramatically change our findings is unclear. Our network tool was inclusive of such documents although they were the minority of articles in both network analyses. Indeed, publishing frameworks outside of journal articles creates challenges, both in terms of physically obtaining the material and being able to grasp the conceptual and operational components dispersed throughout a given textbook. Perhaps corresponding peer-reviewed articles serving as a book review, preferably in open-access formats to improve dissemination, could help mitigate access and citation issues [36].

## Conclusion

In conclusion, bibliometric analysis is one way to understand how D&I frameworks are used in the development of D&I science. We used a bibliometric citation analysis tool to help identify the most prevalent models influencing D&I. D&I researchers and practitioners may consider frequency of citation and this network structure when planning implementation efforts to build upon this foundation and promote systematic advances in D&I science.

## Additional file

Additional file 1: Citation Network Analysis Methods. (DOCX 14 kb)

## Abbreviations

CFIR: Consolidated Framework for Implementation Research
CNA: Citation Network Analyzer
D&I: Dissemination and implementation
KTA: Knowledge to Action Framework
PARiHS: Promoting Action on Research Implementation in Health Services Framework
TDF: Theoretical Domains Framework
