# Supplementary material for: Assessing citation networks for dissemination and implementation research frameworks
Source: Implement Sci. 2017 Jul 28;12:97. doi: 10.1186/s13012-017-0628-2 (PMC5534119; doi:10.1186/s13012-017-0628-2)
Supplement: Additional file 1: — Citation Network Analysis Methods. (DOCX 14 kb) [file 13012_2017_628_MOESM1_ESM.docx]

*Citation Network Analysis Methods Supplement*

*Tabak et al. citation network* *analysis*

For our initial citation network analysis, we used the Tabak review article to serve as the seed for the citation network’s snowball sample. First, we selected the ‘distance’ from this seed article at three levels to capture its broad network relationships across disciplines. These levels specify the hierarchical depth of the network structure: starting with the seed article, the first level specifies those articles citing the seed; the second level specifies those citing the first level; and so on. In most instances, lower levels include citations that follow chronologically from upper levels. However, occasionally, a lower-level publication will cite a source published after it; this is typically due to a citation referencing an earlier release or draft of the publication. While three levels for a larger network might not be parsimonious, we were able to proceed efficiently given there was a single seed article. Therefore, this sampling technique identified level one articles referencing the source article, as well as levels two and three articles with shared citations both after and prior to the Tabak article year of 2012. Second, we selected a 10% rate of data collection at each level to identify only the most central publications within the citation network. The constrained snowball sample selects the top 10% of articles at each level based on their page rank centrality, rather than using a random sample. This is desirable for our goal of identifying major sources in the literature, since it allows us to construct a network consisting of the most prominently cited publications. Last, we used descriptive analyses to examine citation rates, leading journals and authors across the citation network for the Tabak review article.

*Citation network analysis of selected D&I frameworks*

We used data generated from a constrained snowball sample of ten D&I framework articles from the Google Scholar™ academic database selected due to their citation rates and relevance to implementation science. We sampled at a 2% rate and chose one level from each seed article for this part of our study as our ‘distance’, instead of three, due to the number of seed articles and computing capacity limitations. Using this approach allowed for only the most relevant research related to the seed articles while keeping the data collection parsimonious. The remainder of the data collection was similar to that for the Tabak review.
